# Mixture‐Quantitative Structure–Property Relationship Approaches for Jet Fuel‐Component Blends: A Review

**DOI:** 10.1002/minf.70050

**Published:** 2026-08-26

**Authors:** Erik Yeghyan, Benoit Creton, Mickaël Matrat, Gilles Marcou

**Affiliations:** ^1^ IFP Energies nouvelles Rueil‐Malmaison France; ^2^ Laboratoire de Chémoinformatique Université de Strasbourg Strasbourg France

**Keywords:** hybrid model, jet fuels, mixtures, QSPR, SAFs

## Abstract

Jet fuels (JFs) and sustainable aviation fuels (SAFs) are complex hydrocarbon mixtures whose properties must meet strict international specifications to ensure consistent quality. Their increasing variability, driven by environmental and process constraints, raises multiple questions regarding the relationship between fuel composition, safety, and performance. In this context, predictive models can help support fuel formulation while reducing experimental effort. Quantitative structure–property relationship (QSPR) methods have been applied to fuel components such as hydrocarbons to predict various properties, showing promising results but often with limited accuracy and applicability. Several studies have also investigated mixture‐QSPR (M‐QSPR) approaches for hydrocarbon systems involving constituents also present in fuels; however, very few studies directly address real fuel mixtures. This is because M‐QSPR remains particularly challenging due to mixture interactions, data scarcity, representation difficulties, and validation issues. This review evaluates past M‐QSPR studies on fuels, fuel‐component mixtures, and general M‐QSPR methodologies to identify current advances, limitations, and gaps and to outline strategies for developing M‐QSPR models for JFs.

## Graphical abstract

A literature review of QSPR approaches to predict jet fuel properties. It exposes the fundamental principles of QSPR and illustrates pure‐compound QSPR through examples of applications for jet fuel components. Mixture‐QSPR (M‐QSPR) modeling specificities are addressed: mixtures representation, design of descriptors for mixtures, and impact on internal and external validation strategies.

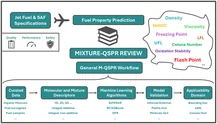

**Abbreviations**
AChE: Acetylcholinesterase
ACSF: Atom‐Centered Symmetry Functions
AD: Applicability Domain
AI: Artificial Intelligence
ANN: Artificial Neural Network
ASNN: Associative Neural Network
ASTM: American Society for Testing and Materials
CN: Cetane Number
CoMFA: Comparative Molecular Field Analysis
COSMOtherm: Conductor‐like Screening Model for Thermodynamics
CV: Cross‐Validation
DFT: Density Functional Theory
ECFP: Extended‐Connectivity Fingerprints
E‐GNN: Equivariant Graph Neural Network
FGCD: Functional Group Count Descriptors
FP: Flash Point
GA: Genetic Algorithm
GA‐MLR: Genetic Algorithm‐Multiple Linear Regression
GC×GC: Comprehensive Two‐Dimensional Gas Chromatography
GETAWAY: GEometry, Topology, and Atom‐Weights AssemblY
GFA: Genetic Function Approximation
GHG: Greenhouse Gas
GNN: Graph Neural Network
GPR: Gaussian Process Regression
GRID: GRid‐INdependent Descriptors
GRIND: GRid‐INdependent Descriptors
InChI: International Chemical Identifier
ISIDA: In SIlico Design and Data Analysis
IUPAC: International Union of Pure and Applied Chemistry
JF: Jet Fuel
kNN: k‐Nearest Neighbors
LFL: Lower Flammability Limit
LOO: Leave‐One‐Out
MACCS: Molecular ACCess System
MAE: Mean Absolute Error
MInChI: Mixture InChI
ML: Machine Learning
MLR: Multiple Linear Regression
MOL: MOL File Format
MON: Motor Octane Number
MPNN: Message‐Passing Neural Network
MSD: Mean Signed Difference
MSE: Mean Squared Error
MWA: Modified Weighted‐Average
PCA: Principal Component Analysis
PFI: Permutation Feature Importance
PLS: Partial Least Squares
QSAR: Quantitative Structure–Activity Relationship
QSPR: Quantitative Structure–Property Relationship
RBF: Radial Basis Function
RBF‐ANN: Radial Basis Function‐Artificial Neural Network
RF: Random Forest
RMSE: Root Mean Squared Error
RON: Research Octane Number
SAF: Sustainable Aviation Fuel
SBC: Synthetic Blending Component
SDF: Structural Data File
SHAP: SHapley Additive exPlanations
SiRMS: Simplex Representation of Molecular Structure
SLT: Superheat Limit Temperature
SMARTS: SMILES Arbitrary Target Specification
SMILES: Simplified Molecular Input Line Entry System
SOAP: Smooth Overlap of Atomic Positions
SSE: Sum of Squared Errors
SVM: Support Vector Machine
SVM‐RBF: Support Vector Machine with Radial Basis Function Kernel
SVR: Support Vector Regression
TPSA: Topological Polar Surface Area
TPRF: Toluene Primary Reference Fuel
UFL: Upper Flammability Limit
UNIFAC: UNIQUAC Functional‐group Activity Coefficient
UNIQUAC: Universal Quasi‐Chemical
WHIM: Weighted Holistic Invariant Molecular Descriptors
XGBoost: eXtreme Gradient Boosting

## 1 Introduction

Fighting global warming, mainly driven by the continuous rise of greenhouse gas (GHG) emissions, represents one of the most pressing challenges of our time. Industrial activities and more specifically the transport sector are among the largest emitters of anthropogenic GHGs, contributing to economic growth while also intensifying climate change. In 2023, transportation alone accounted for 15.8% of global GHG emissions [1], making it one of the sectors where emission reductions would have a significant impact. The aviation sector accounts for about 11% of transportation‐related emissions, corresponding to approximately 2%–2.5% of the worldwide CO_2_ emitted [2, 3]. Given the projected growth of air traffic and the long lifetime of aircraft fleets, it is necessary to rapidly develop low‐carbon fuels to decarbonize this sector. Currently, several technological pathways have been considered, including full electrification, hydrogen propulsion, and the use of alternative liquid fuels [3]. Electrification and hydrogen remain highly challenging due to range and payload constraints, energy density requirements, and infrastructure limitations. In contrast, drop‐in liquid fuels, such as sustainable aviation fuels (SAFs), are already compatible with the existing fleet and supply chain, making them the most realistic near‐ and mid‐term option.

SAFs are blends of conventional jet fuels (JFs) from petroleum and synthetic blending components (SBCs) from renewable resources. Currently, under the ReFuelEU Aviation Regulation, the minimum SAF share is 2% and is scheduled to increase to 20% in 2035 and 70% in 2050 [4]. These complex mixtures are mainly composed of different hydrocarbon families but also contain low‐concentration species, such as additives and impurities. The relative proportions of these families strongly influence key fuel properties, and the final distribution differs from conventional JFs, which typically contain a balanced mix of paraffins/isoparaffins (≈40−60 wt%), naphthenes (≈20−40 wt%), and aromatics (≈15−20 wt%) [5, 6, 7]. On the other hand, SBCs are mainly isoparaffinic [8]. To ensure quality, performance, and safety, both conventional JFs and SAFs are subject to strict ASTM (American Society for Testing and Materials) international specifications (ASTM D1655 [9] for JFs and ASTM D7566 [10] for SAFs containing SBCs) that define acceptable ranges for physicochemical properties and compositional limits. This highlights the need to further investigate SAFs, both to expand certified production routes and to better understand how compositional variability influences critical properties relevant to performance, safety, and logistics. In this regard, computational methods, and in particular machine learning (ML)‐based approaches, have emerged as useful tools to support fuel research by enabling property prediction, formulation guidance, and data‐driven insights. Among these, quantitative structure–property relationship (QSPR) modeling represents a relevant tool, particularly for the prediction of physicochemical properties.

QSPR modeling aims to establish statistical relationships between properties and molecular structures [11]. As an intermediate step, molecular descriptors are used to numerically encode structural and physicochemical information. By learning from existing data, QSPR models can then be applied to predict the properties of compounds not considered during the learning stage. However, these predictions are reliable only for compounds that are sufficiently close to the training set (data on which the model is fitted).

The generalization of models is challenged during QSPR model development through validation steps, typically involving internal and external validations. Both are used to empirically measure model predictivity and whether models remain generalizable beyond the training data.

QSPR models have already been developed to model fuel‐related properties, demonstrating their ability to capture structure–property relationships. A nonexhaustive list of examples includes density and viscosity (e.g., Saldana et al. [12] and Cai et al. [13]), cetane number (e.g., Creton et al. [14, 15]), flash point (e.g., Yao et al. [16, 17]), and oxidation stability (e.g., Venegas‐Reynoso et al. [18]). Additionally, ML‐ and QSPR‐based approaches for predicting fuel ignition and flame properties have been extensively discussed in the review proposed by Üstün et al. [19].

While QSPR has demonstrated good potential for single compounds, conventional fuels as well as SAFs are multicomponent systems, and their properties arise not only from characteristics of individual molecules but also from complex, often nonlinear interactions between them [20].

To address this challenge, QSPR methodologies have been extended to mixtures, giving rise to mixture‐QSPR (M‐QSPR). In this regard, an early review by Muratov et al. [21] summarized the existing developments in M‐QSPR, while a more recent review by Belfield et al. [22] discussed M‐QSPR approaches in the context of toxicity prediction. Both reviews address M‐QSPR applied to biological‐related endpoints, but their discussions of concepts, challenges, and limitations are general and can also be applied to other types of mixtures, for example, fuels. Additionally, beyond these works, other methodological directions have been explored in the context of fuels, including approaches such as Mean‐QSPR (e.g., Hall et al. [23]), composition‐based modeling (e.g., Shao et al. [24]), or the combination of QSPR with mixing rules (e.g., Saldana et al. [17]). Nevertheless, M‐QSPR remains far less developed than QSPR on single compounds. Major challenges include the scarcity of available data and the need for mixture‐level descriptors (in addition to molecular descriptors) that can capture both additive and non‐additive contributions of components [21]. M‐QSPR also requires specific validation strategies, addressing multiple levels of incompleteness of information: Points‐out (unseen ratios), Mixtures‐out (unseen mixtures), Compounds‐out (mixture with at least one novel component), and Everything‐out (entirely unseen systems).

Additionally, both single and mixture compounds contain trace species [25]. These components can have disproportionate, nonlinear impacts on key properties, such as the oxidation stability of JFs [25, 26]. Despite their impact, they are currently not explicitly addressed in QSPR approaches, and to the best of our knowledge, there is a complete lack of dedicated QSPR modeling strategies for them in the literature.

Altogether, the presence of multiple interacting components, heterogeneous data, the need for specific validation schemes, and influential trace species make the modeling of real fuels particularly complex.

In this review, we present methodological concepts and discuss existing studies relevant to understanding and developing M‐QSPR models in the context of aviation fuels. We first provide a concise overview of the main steps involved in conventional QSPR modeling, followed by their extension to mixtures (i.e., M‐QSPR). We then examine how these methods have been applied in existing M‐QSPR studies relevant to fuels. Because direct applications to aviation fuels remain scarce, we enrich the discussion with both organic‐component mixtures and fuel‐component mixtures as defined later. We also briefly discuss alternative approaches to fuel‐property modeling. Overall, this review aims to provide a comprehensive methodological foundation containing the essential information needed to develop M‐QSPR models, particularly in the context of aviation fuels.

## 2 Brief Methodological Background on QSPR Modeling

General QSPR principles have already been extensively reviewed elsewhere [11, 27, 28, 29, 30, 31]. Therefore, this section is intended only as a concise methodological background to introduce the concepts needed for the subsequent discussion of M‐QSPR models and their applications in fuel‐related studies.

The aim of QSPR is to establish a statistical relationship between the chemical structure of a molecule and a given endpoint, assuming that structurally similar compounds have similar endpoint values. The modeling process generally follows a standardized workflow. It includes several key steps such as data collection and preprocessing, calculation and selection of molecular descriptors, definition of an applicability domain (AD) of the model, training of the model using ML algorithms, model validation through internal and external procedures, and assessment using performance metrics.

### 2.1 Data Collection and Curation

In QSPR modeling, the minimum required data include target endpoint values and corresponding chemical structures. Endpoint values can be obtained from various sources including experiments and modeling [27]. Chemical structures are generally encoded into widely accepted electronic formats such as SMILES [32] (Simplified Molecular Input Line Entry System) strings, MOL files, or SDF (Structural Data File) files [33], from which molecular descriptors are computed.

Inconsistencies in collected data may occur, especially when values are derived from different protocols or even different devices and methodologies. Therefore, careful evaluation of data quality is essential to ensure consistency, reproducibility, and completeness [11, 30, 34, 35].

In addition, key curation steps such as removing outliers, handling missing values, and verifying the structural accuracy of compounds are critical for model building [35]. Once the data have been curated, structural standardization is performed to ensure uniformity across datasets, for example, by harmonizing aromaticity representations (Kekulé vs. circle notation) or tautomeric forms [11].

### 2.2 Molecular Descriptors

Molecular descriptors are quantitative (numerical) representations of molecular structures and properties that play an important role in QSPR modeling [11, 34, 36]. These descriptors encode structural, electronic, and physicochemical information, providing the foundation for correlating molecular structures with target properties.

**FIGURE 1:**
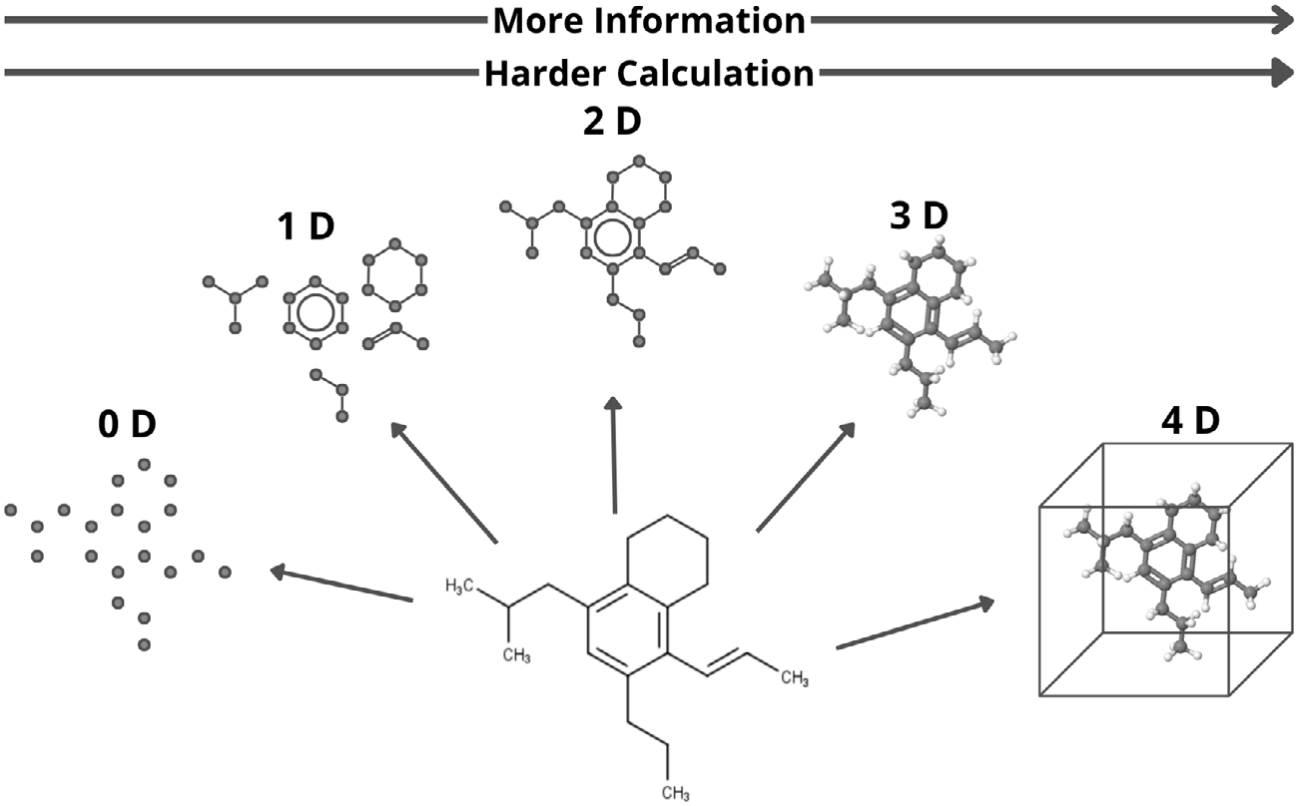
Classification of molecular descriptors from 0D to 4D. Descriptor complexity increases from constitutional descriptors based on molecular formula to 1D fragment descriptors, 2D connectivity descriptors, 3D geometry‐based descriptors, and 4D descriptors incorporating conformational ensembles.

A commonly used classification ranks molecular descriptors from 0D to 4D (Figure 1) [37]. 0D (constitutional) descriptors rely only on the molecular formula; typical examples include atom counts, molecular weight, and elemental composition ratios. 1D descriptors are fragment‐ or sequence‐based, capturing the presence or frequency of predefined substructures or functional groups; these are often encoded as fingerprints such as MACCS (Molecular ACCess System) [38] or predefined SMARTS (SMILES arbitrary target specification) [39]. 2D descriptors are derived from the connectivity graph of the molecule (atoms as nodes, bonds as edges). Some examples of this category include ECFP (Extended‐Connectivity Fingerprints) [40], Wiener index [41], Randić connectivity index [42], χ‐indices, or topological polar surface area (TPSA) [43]. 3D descriptors require an optimized molecular conformation, usually a geometry‐optimized low‐energy structure, and capture spatial and electronic features such as CoMFA (comparative molecular field analysis) [44] steric and electrostatic fields, GRID/GRid‐INdependent Descriptors (GRIND) [45, 46] interaction fields, and VolSurf [47] descriptors, 3D‐MoRSE (Molecule Representation of Structures based on Electron diffraction) [48], WHIM (Weighted Holistic Invariant Molecular descriptors) [49], and GETAWAY (GEometry, Topology, and Atom‐Weights AssemblY) indices [50], atom‐centered symmetry functions (ACSF) [51], smooth overlap of atomic positions (SOAP) [52], solvent‐accessible surface area, molecular volume, or dipole moment. Finally, 4D descriptors extend this concept to conformational ensembles, incorporating multiple conformations, orientations, or time‐dependent behavior. An example is the extension of COSMOtherm (Conductor‐like Screening Model for Thermodynamics) to multiple conformers [53].

The generation and selection of descriptors are critical steps to ensure that the most relevant and informative features are used in the modeling process to describe the chemical structure [11, 31, 34]. However, with thousands of possible descriptors available and a limited number of instances, variable selection can induce Freedman's paradox [54], i.e., modeling noise with noise. This implies that the variable selection procedure must be integrated within the modeling and validation workflow. The variable selection can be implicit in an ML algorithm (such as embedding approaches) [55]. Otherwise an optimization strategy is used with the aim of improving the model's performance such as a genetic algorithm (GA) [56]. Molecular descriptors are also frequently filtered out because they are highly correlated with one another or nearly constant. Yet, these decisions are based on a finite number of instances, the training set, and are not going to generalize to new instances, a priori. For this reason, they must be considered with care as they are part of the definition of the molecules that are relevant for using the QSPR model.

Beyond conventional handcrafted descriptors, recent artificial intelligence (AI)‐driven molecular representations have emerged as alternatives for molecular property prediction. Chemical language models, such as ChemBERTa [57] (ArXiv), learn molecular embeddings directly from string representations (e.g., SMILES) using transformer architectures originally introduced for sequence modeling [58] and self‐supervised pretraining. Graph neural networks (GNNs), including message‐passing neural networks (MPNNs) [59], instead learn representations directly from molecular graphs. More recently, equivariant GNNs (E‐GNNs) [60] have extended this idea to three‐dimensional molecular structures by preserving the symmetry of molecular properties with respect to rotations, translations, and reflections. These approaches are not conventional descriptors in the strict sense, but learned molecular embeddings that can complement or replace predefined descriptor lists.

### 2.3 Chemical Space and AD

Chemical space is the theoretical universe of all conceivable molecules. Bohacek et al. [61] estimated that by assembling up to 30 C, N, O, or S atoms, the theoretical chemical space could contain more than 10^60^ possible structures. In practice, only a tiny fraction of this space is sampled, with each molecule in a dataset represented as a point in an n‐dimensional descriptor space (one axis per descriptor).

**FIGURE 2:**
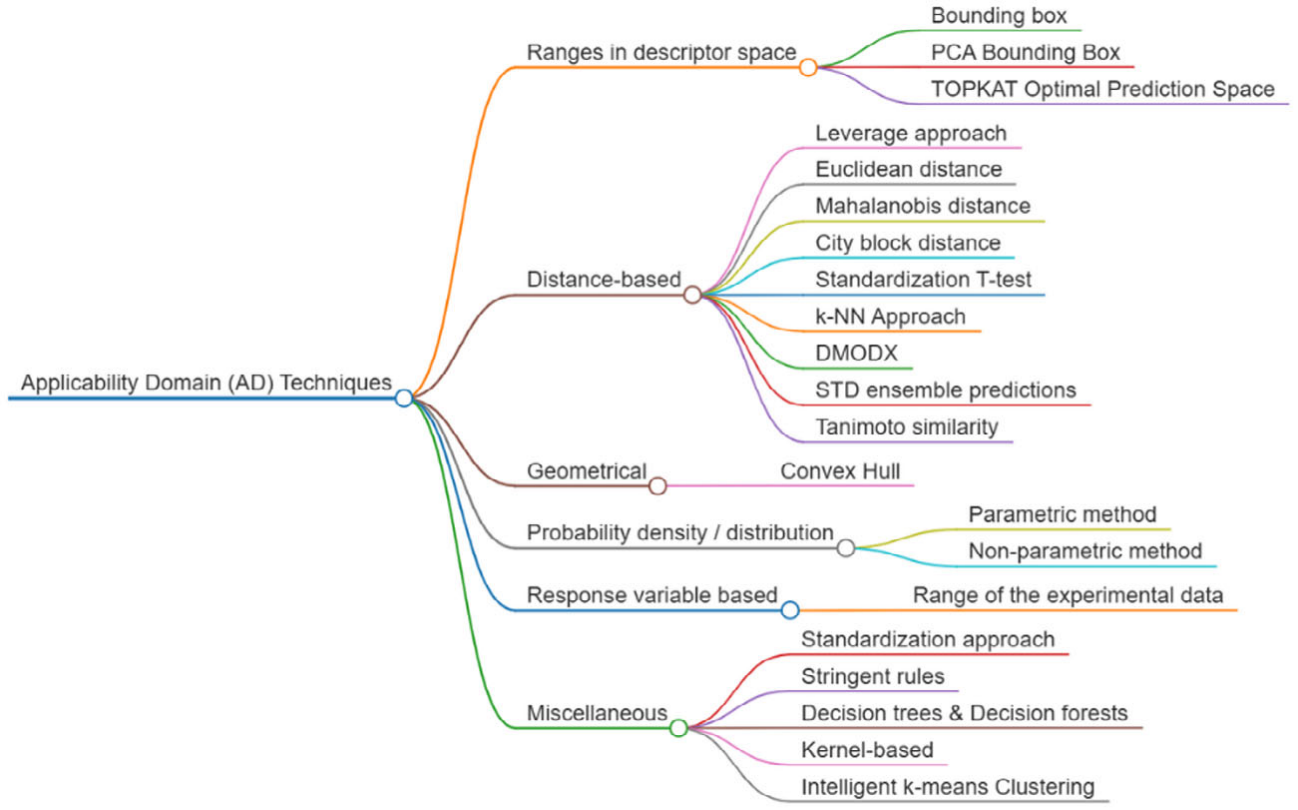
An ontology of applicability domain approaches (adapted from reference [63]).

Dimensionality‐reduction techniques (e.g., principal component analysis (PCA) [62]) are used to summarize the dataset on one‐, two‐, three‐, or even four‐dimensional plots. The resulting low‐dimensional maps help to illustrate trends and clusters, identify gaps, flag outliers, and develop chemical intuition about the dataset. The AD of a model defines the portion of the chemical space where the model's predictions can be considered reliable. Establishing an AD is essential because a model is only valid for chemicals similar to those it was trained on [11, 27, 34, 63, 64]. If a new molecule is outside of the AD, the prediction becomes a model extrapolation and should be considered uncertain: it is no longer possible to control the prediction error of the model. The AD can be determined using various approaches based on descriptors or the given endpoint values. These include range‐based techniques in descriptor space, distance‐based approaches, geometrical methods, probability density distribution models, response variable‐based techniques, and other miscellaneous strategies, as illustrated in Figure 2 [63].

**FIGURE 3:**
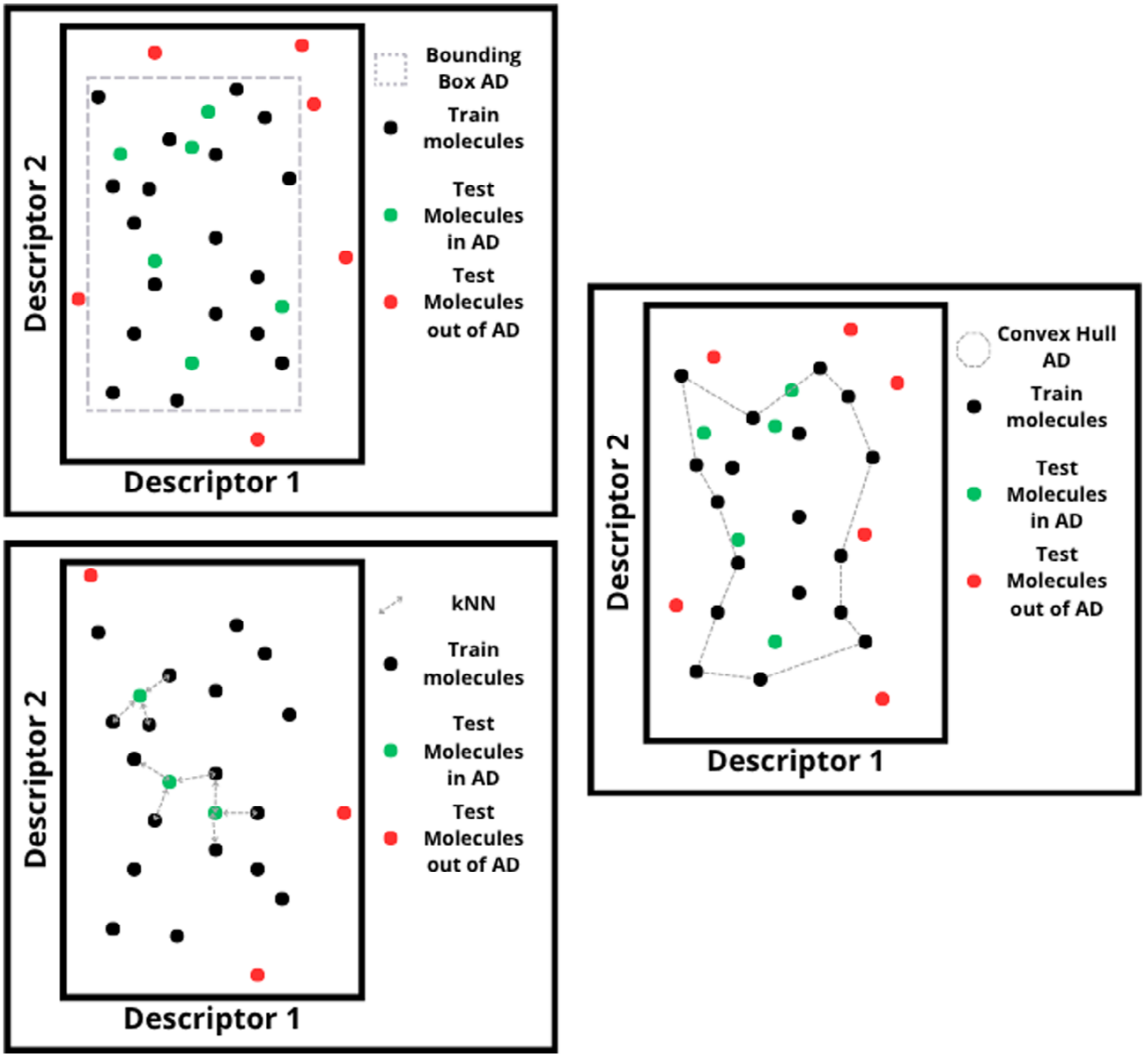
Examples of geometrical applicability domain (AD) approaches, including the bounding box, *k*‐nearest neighbors (kNN), and convex hull methods (adapted from reference [63]).

Some examples of geometrical AD approaches are shown in Figure 3 [63, 64]. The bounding box method is an intuitive definition of the AD: a new compound is inside the AD if the value of each molecular descriptor is within a predefined range, usually the minimal and maximal values observed in the training set. The *k*‐nearest neighbors (kNN) method considers a compound within the AD if its distance to neighboring training compounds is below a predefined threshold, while the convex hull method defines the AD as the smallest convex region enclosing the training compounds. Compounds located outside these boundaries are considered outside of the AD.

### 2.4 Performance Metrics and Training of ML Algorithms

Since fuel‐related QSPR studies most frequently implement regression tasks, as most fuel properties are continuous variables, this section focuses on supervised ML algorithms for regression. The aim of such algorithms is to optimize the fit of the model to the training data, as measured by a loss function. The most commonly used loss function is the sum of squared errors (SSE) [65]. However, other performance metrics such as the coefficient of determination (*R* ^2^), cross‐validated *R* ^2^ (*Q* ^2^), mean absolute error (MAE), mean squared error (MSE), root mean squared error (RMSE), and mean signed difference or bias (MSD) are commonly reported to facilitate interpretation and comparison of model performance [31, 34, 65].

QSPR models can be derived by applying different ML algorithms, each based on different approaches, such as linear methods, analogizers, symbolists, and connectionists [66].

One of the most interpretable algorithms is multiple linear regression (MLR) [67], which fits a linear relation between the property of interest and the molecular descriptors. In MLR, each coefficient indicates the contribution of a descriptor to the prediction, making the model easy to interpret provided that descriptor values were standardized. The main assumption of this approach is that the effect of each descriptor is additive and independent. A recent benchmark by Wu et al. [66] compared the performance of 16 different ML algorithms across 14 biochemical datasets. They found that radial basis function support vector machine (SVM‐RBF) [68], XGBoost [69], and Gaussian process regression (GPR) [70] consistently outperformed others, highlighting them as top choices for accurate and reliable QSPR modeling. However, the no free lunch theorem [71] should caution against drawing definitive conclusions following a specific benchmark. If one ML algorithm performs well, others may also perform well under appropriate conditions.

SVMs [68] are widely used in QSPR [15, 18, 72, 73]. In regression tasks, Support vector regression (SVR) uses an ε‐insensitive loss function: prediction errors within an ε‐wide tolerance region are not penalized, whereas larger deviations contribute to the loss. This has two major benefits: the model does not adjust data better than a user‐defined precision and the model is more robust toward outliers. The solution is based on a subset of the training data points, the support vectors. Thus, the model is also robust to perturbations of the training set. Through kernel functions, such as the radial basis function, SVMs can capture nonlinear relationships by implicitly mapping descriptors into a higher dimensional space. SVM models are valued in QSPR studies for their robustness, ability to handle complex relationships, and capacity to avoid overfitting through regularization, often achieving strong performance even on small datasets. The explainability of such a model can be unintuitive, but post hoc methods such as SHAP (SHapley Additive exPlanations) [74] can provide insights into the contribution of individual descriptors to the model output, as in Venegas‐Reynoso's study [18]. Other model‐agnostic interpretability approaches, such as permutation feature importance (PFI), can also be employed to assess descriptor relevance by quantifying the decrease in predictive performance resulting from the random shuffling of individual variable values [75]. It should be noted that these interpretability techniques are broadly applicable to other nonlinear and ensemble‐based models, including random forests (RF), gradient boosting, and neural networks.

Decision trees [76] and ensemble methods represent another important class of algorithms. A single decision tree partitions data based on descriptor value thresholds, generating a hierarchy of decision rules. While individual trees may be unstable, ensemble strategies substantially improve predictive performance. RF [77] construct numerous trees on random subsets of the dataset and then average their predictions, reducing variance and improving robustness. These models handle nonlinear effects and interactions naturally, and they provide estimates of feature importance. Gradient boosting methods such as XGBoost [69], on the other hand, build trees sequentially, with each new tree correcting the residual of the previous one. Such boosting approaches are often highly accurate, though less interpretable than simple trees, and their complexity requires careful inspection of variable importance or partial dependence plots.

GPR [70] represents another family of algorithms based on probabilistic distribution learning. In contrast to SVMs, GPR is intrinsically kernel‐based, as the covariance function defines the prior over functions. GPR models the relationship between molecular descriptors and target properties within a nonparametric Bayesian framework, where a prior distribution is placed over possible functions, enabling both accurate predictions and estimation of predictive uncertainty. GPR captures complex nonlinear relationships through covariance kernels such as the radial basis function. It is a method of choice to obtain a self‐consistent predicted value and an estimated error of prediction. Because the output of GPR is a probability density, GPR can also be useful as a building block for generative models. However, its computational cost for training increases as *N* ^3^ with the number *N* of *D*‐dimensional training samples, so it is better suited for small datasets or very low dimensional problems [70, 78].

Finally, the family of artificial neural networks (ANN) [79] has become increasingly relevant for QSPR applications. ANNs consist of layers of interconnected computing units capable of learning arbitrary nonlinear mappings given sufficient data. In a typical QSPR setting, descriptor values are fed into the input layer, propagated through one or more hidden layers with nonlinear activation functions, and then mapped to the predicted property at the output layer. Training involves adjusting connection weights using algorithms such as backpropagation [80] to minimize prediction error. These models can capture complex, high‐order interactions. The learning process is based on batches of data, so it can be trained on very large datasets. The learning procedure has no defined end so the training of an artificial neural network can be restarted any time. The adaptive structure of the model allows contextual changes to be integrated in the training data, for instance, for pretraining and fine‐tuning of the models. However, ANNs are often considered black‐box models; they may suffer from convergence instability and overfitting, typically rely on empirical regularization techniques (e.g., dropout or early stopping), and generally require larger training datasets than classical ML methods to achieve robust generalization.

### 2.5 Model Validation

**FIGURE 4:**
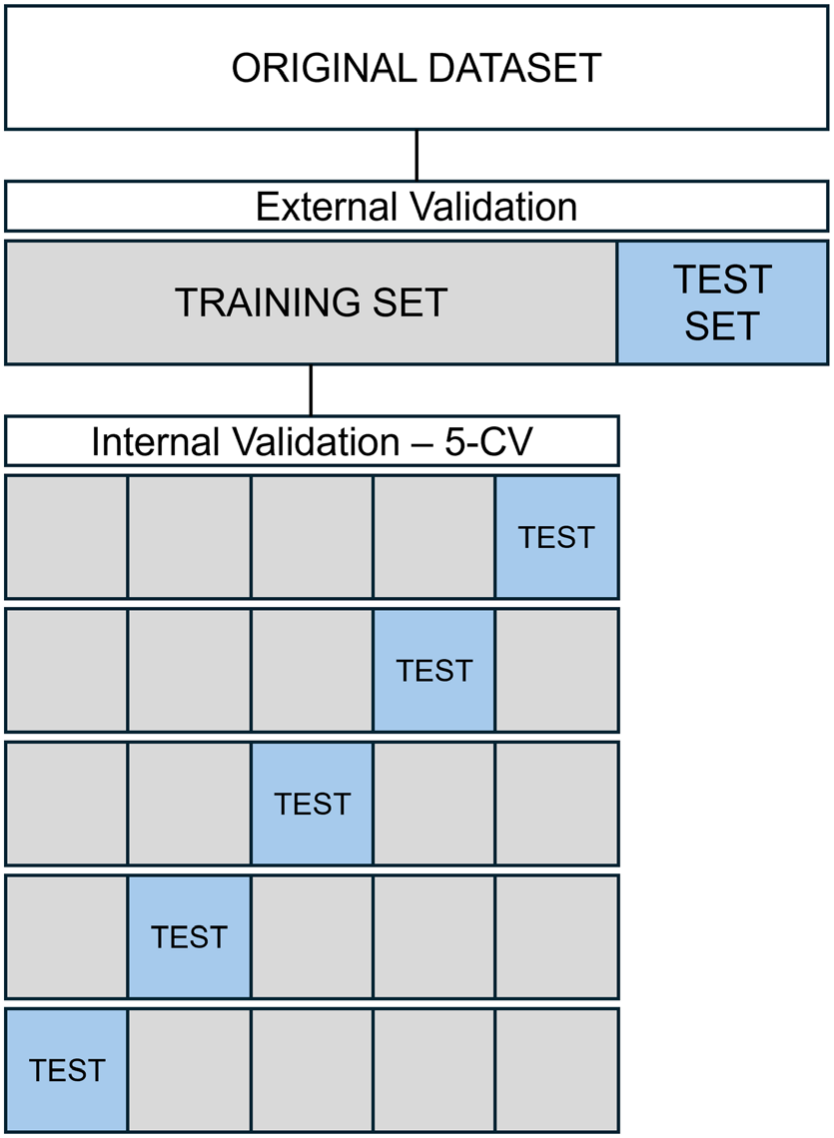
Schematic representation of an external train/test split and an internal 5‐fold cross‐validation.

The validation step in the QSPR modeling workflow assesses how well the model explains the training data and generalizes to unseen data [11, 27, 34, 81]. This process involves testing the model's performance on both the training dataset (internal validation) and an independent dataset (external validation). Validation is not only essential for assessing the model's predictive capability but also for its acceptance in scientific and regulatory contexts [34, 82]. Internal validation involves testing the model on subsets of the training data that were not seen during model fitting on the train set. A common approach is *k*‐fold cross‐validation (*k*‐fold CV), where the dataset is split into *k* parts, the model is trained on *k*–1 parts and tested on the remaining part, and this process is repeated *k* times so each portion serves as a test once. When *k* equals the number of instances in the dataset, it is called Leave‐One‐Out (LOO) cross‐validation: each compound is left out once individually to be tested and the remaining ones serve as the training set [31, 34]. Internal validation is used to monitor how a model fits both the training and test data. This helps optimize modeling choices (e.g., hyperparameter values) that are likely to lead to models that are neither underfitted nor overfitted to the dataset [31, 34]. The outcome is often summarized by metrics like *Q* ^2^ (the cross‐validated *R* ^2^) which indicates how well the model predicts left‐out data. A model with a high training *R* ^2^ but low *Q* ^2^ is considered overfitted [11, 31, 34] (memorizing training data rather than capturing general patterns). External validation involves testing the model on an independent dataset of compounds that played no role during model training or model selection [11, 31, 34]. External validation simulates real‐world prediction scenarios. Performance on this held‐out test set provides an unbiased estimate of how the model will generalize to truly new chemicals. External validation is crucial because it challenges the entire prediction process: standardization of query molecules, calculation of the molecular descriptors, AD, and quality of the ML model. Poor external performance may indicate limitations in the modeling workflow, such as insufficient data curation, inappropriate descriptors, overfitting, or an inadequate AD definition. It may also indicate that the external compounds are outside the chemical domain covered by the training set [31, 34, 64]. Figure 4 provides a schematic representation of external and internal validation procedures. In this example, the original dataset is first split into external training and test sets for external validation, and the external training set is subsequently used to perform an internal 5‐fold cross‐validation.

Moreover, nested cross‐validation can also be employed for model validation when model selection or hyperparameter optimization is required. In this scheme, an outer *k*‐fold cross‐validation is used to estimate predictive performance, whereas an inner *i*‐fold cross‐validation is performed within each training fold to optimize hyperparameters [18, 83]. This strategy reduces the optimistic bias that can occur when the same data are used for both hyperparameter optimization and performance estimation because model selection is repeated inside each outer training fold [83].

### 2.6 QSPR Study for JF Components—A Typical Case Study

To illustrate the above discussion, we selected a recent QSPR study that follows the principal steps described above. This work of Venegas‐Reynoso et al. [18, 84] focuses on the modeling of the oxidation stability of pure hydrocarbons present in SAFs and JFs. The oxidation of the fuel can alter its composition and properties, leading to performance issues and potential system failures. The work is based on a dataset of more than 200 experimental measurements of oxidation stability covering diverse molecular families including n‐, iso‐, cyclic, aromatic, and olefinic hydrocarbons measured using the standardized RapidOxy method [85].

Molecular structures were encoded into 1D/2D functional‐group count descriptors (FGCD) derived from SMARTS patterns. These descriptors are a type of 2D molecular descriptor as described above in Section 2.2, with the experimental temperature included as an additional descriptor.

The authors used SVM‐RBF and XGBoost algorithms and validated their models using a nested cross‐validation scheme with 4‐fold external and 10‐fold internal loops for parameter optimization. The most promising models (on log‐transformed endpoint values) were obtained with XGBoost. The authors also used a data augmentation strategy contributing to improve their models. However, the average relative error (48.6%) was substantially higher than the experimental repeatability of 10.8%. This observation supports using the models in their current state for semiquantitative reliability and preliminary screening. It also calls for more detailed analysis of observed mispredictions. For instance, reported purity of the compounds used in the experiments ranged from approximately 90% to 99.9%, implying potentially non‐negligible amounts of impurities that are known to significantly influence oxidation stability [25].

## 3 M‐QSPR for Hydrocarbons and Fuel‐Relevant Systems

Since fuels are multicomponent systems, classical QSPR must be extended to M‐QSPR. This shift, for instance, requires the introduction of mixture‐level descriptors, complementing molecular ones, to represent both additive and nonadditive mixture behavior. Moreover, dedicated validation schemes are needed to ensure predictive reliability across unseen ratios, mixtures, or compounds.

The following section first outlines the general methodological principles of M‐QSPR and then discusses existing studies applying M‐QSPR to fuel‐related mixtures.

### 3.1 Mixture Data in M‐QSPR

Most M‐QSPR studies have been based on binary or ternary mixtures [21], as these systems are experimentally easier to characterize and computationally simpler to model, with each blending component being clearly identifiable.

In practice, a mixture can be described by listing each component's structure together with its proportion (mole, mass, or volume fraction). One simple convention is the use of dot‐disconnected SMILES, in which multiple molecular SMILES are concatenated with a “.” to denote separate constituents. While straightforward, this notation omits compositional information such as the relative fractions of each component.

**FIGURE 5:**
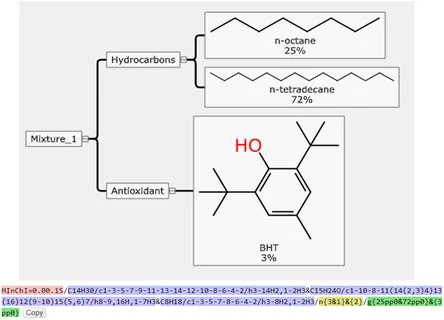
Example of a Mixfile and the corresponding MInChI (adapted from the MInChI demo tool) [87].

An alternative is to use multimolecule structure files, for instance, an MDL Molfile or SDF containing several structures to describe a mixture. However, these representations are not standardized, and component proportions are typically stored in data fields attached to the structures. McEwen et al. [86] introduced the Mixfile format, a machine‐readable standard designed to encode multiple components and their respective proportions within a single file. The International Union of Pure and Applied Chemistry (IUPAC) recommends the use of Mixture InChI (MInChI) [86], an extension of the InChI identifier scheme that allows unambiguous digital representation of mixtures (Figure 5).

Finally, a mixture can be treated as a record in a database where component identifiers (e.g., SMILES) and their corresponding fractions are listed in a table. For example, in the study by Creton et al. [15], fuel surrogate mixtures were encoded in a matrix with mixtures as rows and all possible components and their volumetric fractions as columns.

### 3.2 Mixture Descriptors in M‐QSPR

Integral additive descriptors remain the most widely used in M‐QSPR due to their simplicity and low computational cost [11, 21, 22]. They are derived by combining the descriptors of individual components (*D*_*i*_) often weighted by their mole, mass, or volume fractions (*x*_*i*_). However, such descriptors do not explicitly capture all the intermolecular interactions present in mixtures.

Several mathematical formulas have been discussed in the literature to define additive mixture descriptors. Table 1 summarizes the most commonly used expressions and their corresponding references. In a toxicity‐related study [88, 96], the Cubic Ratio Norm and Square Descriptor formulas showed the highest predictive performance. Additionally, Mahini et al. [89] recently proposed a Python package to calculate some of those additive descriptors for modeling.

**TABLE 1:** Mixing rules proposed to derive mixture descriptors based on their individual component descriptors (*D*_*i*_) and fractions (*x*_*i*_).

Type	Formula
Weighted average (Kay's mixing rule) [15, 16, 17, 72, 88, 89, 90, 91, 92, 93, 94]	$D_{mix}=\sum_{i=1}^{n}x_{i}*D_{i}$
Arithmetic mean [89, 90, 92, 95]	$D_{mix}=\frac{\sum_{i=1}^{n}D_{i}}{n}$
Square fraction contribution [88, 89, 90, 92, 95]	$D_{mix}={\left(\sum_{i=1}^{n}x_{i}*D_{i}\right)}^{2}$
Norm of fraction contribution [89, 90]	$D_{mix}=\sqrt{\sum_{i=1}^{n}{\left(x_{i}*D_{i}\right)}^{2}}$
Square root fraction [89, 90]	$D_{mix}=\sum_{i=1}^{n}\sqrt{x_{i}}*D_{i}$
Square root descriptor [88, 92]	$D_{mix}=\sum_{i=1}^{n}x_{i}*\sqrt{D_{i}}$
Square descriptor [88, 92, 96]	$D_{mix}=\sum_{i=1}^{n}x_{i}*{\left(D_{i}\right)}^{2}$
Square fraction [89, 90, 92, 95]	$D_{mix}=\sum_{i=1}^{n}{\left(x_{i}\right)}^{2}*D_{i}$
Cube root [92, 97]	$D_{mix}=\sqrt[{3}]{\sum_{i=1}^{n}x_{i}*{\left(D_{i}\right)}^{3}}$
Cubic Ratio Norm [88, 96]	$D_{mix}=\sqrt[{3}]{\sum_{i=1}^{n}{\left(x_{i}\right)}^{3}*D_{i}}$
Weighted difference [72, 92, 95]	$D_{mix}=|x_{1}*D_{1}-x_{2}*D_{2}|$
Independent action [90]	$D_{mix}=\sum_{i=1}^{n}x_{i}*D_{i}-\prod_{i=1}^{n}x_{i}*D_{i}$

On the other hand, there are integral nonadditive descriptors [21], which treat the mixture “as a whole” rather than as a linear combination of independent parts. In practice, integral nonadditive descriptors are often designed to account for specific intermolecular interactions such as hydrogen bonding, dipole–dipole forces, etc. or are computed by considering all components together in one computational model. An example is the descriptor set introduced by Ajmani et al. [21, 94] for binary liquid mixtures. They defined interaction‐sensitive descriptors such as a hydrogen bonding term and a dipole–dipole term for the mixture. The hydrogen‐bond descriptor was formulated using the difference in hydrogen bond donor/acceptor counts of the two components. They found that the H‐bond interaction term had the largest effect on excess volume and mixture density, more than the dipole term. In practice, they quantified hydrogen‐bonding by comparing the donor (D) and acceptor (A) counts of each component and weighting these by their respective mole fractions. The resulting mixture descriptor is defined by Equation (1), where *x*_1_ and *x*_2_ are the mole fractions and *A*_1_, *A*_2_/*D*_1_, *D*_2_ are the acceptor/donor counts for each component. This captures both the mixture composition and the potential for cross hydrogen bonding between dissimilar donors and acceptors.

(1) $$D_{H_{\text{bond}}}=\;x_{1}x_{2}(\;2|A_{1}-\;A_{2}|+\;2|D_{1}-\;D_{2}|-\;A_{1}D_{2}-\;D_{1}A_{2})$$

Another example is the use of combined quantum‐chemical calculations on the mixture, Zhang et al. [21, 98] pooled two molecules together in an ab initio calculation to derive mixture descriptors like the total energy of the dimer against the sum of energies of monomers (as an interaction energy). For a binary mixture they calculated a total energy difference whose value indicates nonadditive interaction energy (stabilization or destabilization when the molecules are mixed).

Another nonadditive descriptor‐calculation method (CombinatorixPy) was proposed by Mahini et al. [96], who recently introduced a Python package that advances the generation of mixture descriptors using a cartesian‐based combinatorial approach. This tool systematically enumerates all possible interactions between components by computing the Cartesian product of descriptor sets for all mixture constituents, thereby generating higher‐order interaction terms beyond simple pairwise combinations. The resulting combinatorial descriptors may capture complex non‐additive interactions that traditional additive mixture descriptors cannot represent. Moreover, in benchmarking studies by the authors [96], models built on combinatorial descriptors outperformed those relying on additive formulations, highlighting the potential of this approach to describe mixtures.

In the same scope, fragment‐based descriptors can also be used as additive descriptors or as nonadditive descriptors. The latter incorporate interactions by considering combined fragments spanning different molecules in the mixture [21]. Nonadditive fragment descriptors are a more sophisticated approach compared to additive ones, as the structural fragments are not limited to those within individual components but they can include “mixed” fragments composed of parts of different molecules. The mixture is treated as one set of molecules in which certain fragments can be formed by picking atoms from different components. SiRMS (simplex representation of molecular structure) [99] is a key example of this approach. In the SiRMS method, molecules are described by simplexes (fragments), which are connected subgraphs of the molecular structure. To adapt this to mixtures, Kuz’min et al. [100] introduced the concept of unbounded simplexes that span two molecules. For a racemic mixture (1:1 of R and S enantiomers), they allowed simplexes where part of the fragment comes from the R‐molecule and part from the S‐molecule. These “mixed‐molecule” fragments exist only in the combined system (the racemate) and not in either pure enantiomer alone. Such descriptors are unique to the mixture and can capture stereochemical interactions or complementarity between the two components. In their acetylcholinesterase (AChE) inhibition study of chiral organophosphates [101], this double 2.5D simplex approach (so called because it considered 2D topology plus a chiral indicator as “half” of 3D) provided descriptors that reflect synergistic or antagonistic effects between enantiomers. Muratov et al. [102] later generalized the SiRMS method to binary mixtures with variable component ratios and applied it to antiviral drug combinations, reporting that models based on SiRMS mixture descriptors outperformed those using only additive descriptors.

Generally, during fragment enumeration, bounded simplexes describe fragments entirely within one component, while unbounded simplexes describe fragments containing atoms from different components. Additionally, a weighting scheme accounts for the mixture ratios, bounded simplex‐based descriptors (D_mix∖b_) are calculated using the classical additive method (Equation (2)), while unbounded simplex‐based descriptors (D_mix∖u_) are determined by weighting the unbound simplex fragment by twice the fraction of the component with the lowest proportion from which the unbounded simplexes were created (Equation (3)). For example, consider a binary mixture A–B with *x*_A_ < *x*_B_.

(2) $$D_{\mathrm{mix}\setminus b}=x_{A}{D_{A}}_{\text{bounded}}+x_{B}{D_{B}}_{\text{bounded}}$$

(3) $$D_{\mathrm{mix}\setminus u}=2x_{A}*{D_{A+B}}_{\text{unbounded}}$$

where *x*_A_ and *x*_B_ are mole fractions of A and B, D_Abounded_ and D_Bbounded_ are simplex molecular descriptors of A and B, and D_A + Bbounded_ is the shared simplex molecular descriptor between A and B.

With this approach, every mixture yields a set of descriptors that includes both “individual fragments” (additive) and “mixed fragments” (nonadditive) encoding interactions.

Another example is the study in which SiRMS and ISIDA (in sIlico design and data analysis) fragment descriptors [103] were used by Oprisiu et al. to model bubble‐point temperatures of binary mixtures [21, 72]. ISIDA descriptors allow to generate a large pool of fragment descriptors, capturing various possible interactions at the fragment level. In this study, unbounded simplexes were used as a nonadditive approach, and bounded simplexes and ISIDA Fragments were used as additive descriptors using previously mentioned methods. Finally, in another study, Yao et al. [16] used nonadditive SiRMS descriptors to model the flash points (FPs) of binary mixtures; they tried nonadditive descriptors because following Saldana et al. [17] the additive descriptors had previously not yielded good results. In support of this, there is a GitHub package that enables users to calculate SiRMS for single compounds, quasimixtures, mixtures, and reactions [104].

Alongside these mixture descriptors, learned molecular representations offer a complementary route for mixture encoding, as illustrated by recent graph‐based mixture‐property models such as MolPool [105] and MolSets [106]. In these approaches, each component is encoded as a molecular graph embedding, which is then combined into a mixture‐level representation using composition‐aware or set‐based pooling. More generally, similar strategies could in principle be extended to other learned molecular embeddings, such as those generated by chemical language models [57], message‐passing GNNs [59], or equivariant GNNs [60]. However, their application in fuels remains emerging and still limited, with MolPool [105] representing one recent fuel‐mixture demonstration, while broader applications across fuel‐related properties remain largely unexplored.

### 3.3 Validation Approaches in M‐QSPR

Mixtures present specific challenges in how information can leak from the training set to the test set during validation [11, 21, 72]. For instance, a validation procedure may decide whether to allow shared components between the training and test mixtures. This decision creates different validation scenarios with distinct performance outcomes. To address this issue, four validation methods have been proposed to simulate different real‐world prediction scenarios, such as Points‐out, Mixtures‐out, Compounds‐out, and Everything‐out validations [21, 22, 72, 94, 107].

Points‐out validation is the least restrictive and easiest to implement. According to this approach, individual data points, typically specific mixture compositions, are left out for testing. Essentially, for a given pair of components that form a binary mixture, one or more data points with particular ratios are set aside, while other data points for that same mixture remain in training. This strategy is only applicable if the dataset contains multiple measurements per mixture (e.g., a binary pair measured at several compositions). We emphasize that with this strategy, the mixture components that are in the test set are not new to the model, only the exact ratio (data point) is new. This assesses the model's ability to interpolate within a familiar system.

**FIGURE 6:**
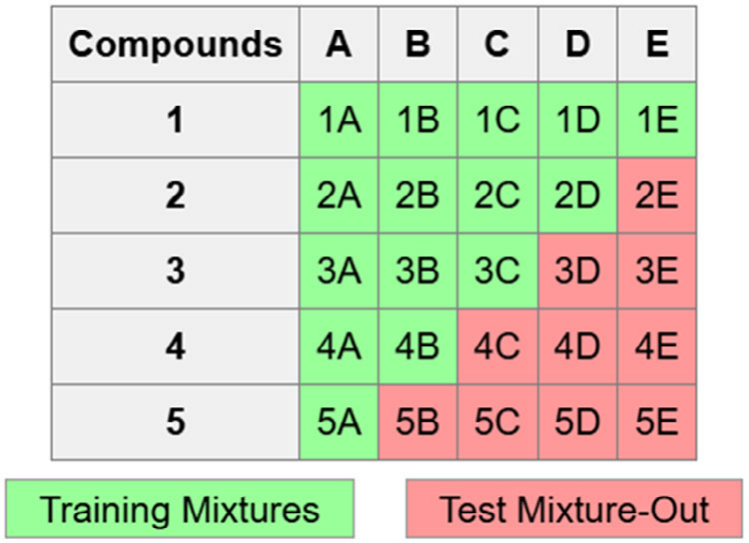
Schematic example of Mixtures‐out validation split between training (green) and test mixtures (red).

In Mixtures‐out validation, entire mixtures are left out from training, which means that all data points corresponding to a particular combination of components are set aside for testing. Figure 6 is a schematic illustration where each cell represents a binary mixture formed by two compounds from the series (1–5) and (A–E). Cells in green correspond to mixtures in the training set, whereas those in red are entirely excluded from the training phase and serve as the Mixtures‐out test set. For instance, the mixture “5E” is held out, meaning that all data points associated with this combination are reserved for testing. This validation simulates predicting properties for a new combination of already known chemicals. Compared to Points‐out, Mixtures‐out validation provides a more realistic evaluation of model generalization to unseen mixture combinations. However, Mixtures‐out evaluation is still limited to the domain of the chemicals in the modeling set without any information about its ability to predict new chemicals.

**FIGURE 7:**
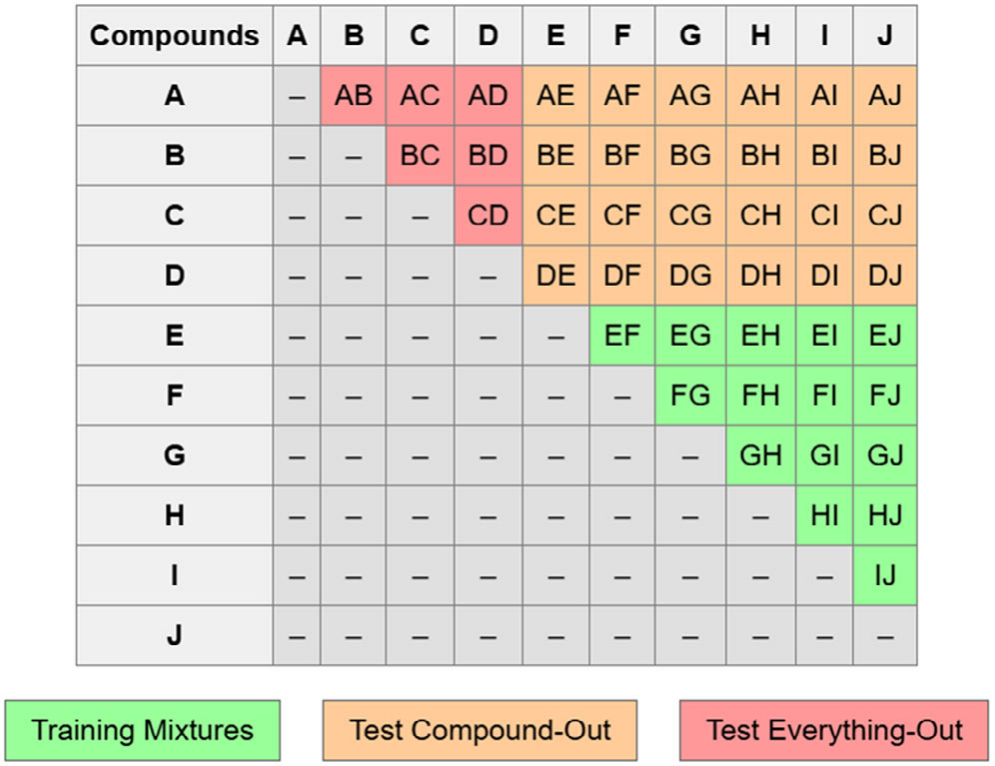
Schematic representation of Compounds‐out and Everything‐out validation strategies for binary mixtures, showing the split between training (green) and test mixtures (orange and red). Compounds A–D are held out for testing, while compounds E–J are used for training.

Compounds‐out validation provides a more stringent assessment of a model's ability to generalize to both unseen compounds and mixtures. Figure 7 illustrates the Compounds‐out validation strategy using a schematic matrix, where each cell represents a binary mixture. Mixtures highlighted in green (with components E to J) correspond to the training set. The orange cells denote the Compounds‐out test region, containing mixtures that include at least one compound absent from the training data—in this case, compounds A–D.

Every test mixture thus includes at least one unseen component, providing a direct measure of the model's ability to predict mixtures containing new chemical entities. Compounds‐out is more rigorous than Mixtures‐out (or Points‐out) validation because the model cannot rely exclusively on previously encountered constituents since every test mixture contains at least one unseen component.

Lastly, the Everything‐out strategy represents the most restrictive validation scenario, designed to evaluate a model's ability to predict the property of mixtures composed entirely of new compounds not included in the training set. In the Everything‐out approach, a subset of compounds is completely excluded from training, and only mixtures formed exclusively from these excluded compounds are used for testing (red cells in Figure 7). In their study, Varlamova et al. demonstrated that the Everything‐out validation approach produced prediction errors comparable to the Compounds‐out strategy [108]. However, its implementation presents practical challenges for small datasets, where the Everything‐out test set can become very small, reducing confidence in performance estimates. Furthermore, for systems involving ternary or higher‐order mixtures, leaving out several components simultaneously may drastically reduce the training dataset size and make this strategy infeasible. Despite these limitations, the Everything‐out validation remains a powerful stress test. If an M‐QSPR model maintains reasonable accuracy under this scheme, it provides strong evidence of the model's ability to generalize to completely unseen chemical systems.

### 3.4 Existing M‐QSPR Studies Applied to Organic‐Component and Fuel‐Component Mixtures

Early M‐QSPR studies that can be considered relevant to JF modeling, mostly investigated low‐component organic mixtures composed of hydrocarbons and heteroatomic species, and typically target thermodynamic or safety‐related properties. Although these early studies were not designed for aviation fuels, they remain informative because they apply M‐QSPR methodologies to chemical families also present in JFs: hydrocarbons as the main constituents and functionalized species present at trace levels. Other M‐QSPR studies are more directly connected to fuels and fuel‐component mixtures and investigate properties explicitly relevant to fuels and JFs.

The following subsections review QSPR modeling for “organic‐component mixture” studies and studies directly involving “fuel‐component mixtures” while also briefly highlighting complementary modeling strategies proposed in the literature. Here, “organic‐component mixture” refers to a mixture of hydrocarbons and heteroatomic species, including, in some cases, systems with fuel‐relevant combinations such as hydrocarbon/hydrocarbon mixtures. In contrast, “fuel‐component mixture” refers to a mixture related to fuel applications, such as surrogate fuels, model blends, or real fuels. These works outline the methodologies that can serve as a foundation for the development of M‐QSPR models applicable to JF‐related systems. Table S1, as provided in the Supporting Information, proposes a summary of M‐QSPR studies relevant to JF‐related systems.

#### 3.4.1 M‐QSPR Applied to Organic‐Component Mixtures

Early M‐QSPR studies by Ajmani et al. [94, 109, 110] illustrated some of the first attempts to extend single‐molecule QSPR concepts to mixtures, and their approach then evolved across three publications. In their first study, authors modeled density deviation for a large and chemically diverse set of binary liquid mixtures using additive descriptors, obtained by simple mole‐fraction averaging of over a thousand molecular descriptors [94]. Ensemble neural‐network models achieved high predictive performance, particularly under Points‐out and Mixtures‐out validation schemes. However, the authors explicitly recognized that this descriptor construction does not encode intermolecular interactions, also limiting the model's ability to generalize beyond the chemical space represented by their dataset.

To address this limitation, Ajmani et al. [109] introduced nonadditive mixture descriptors designed to reflect key interaction drivers such as hydrogen bonding, dipolar interactions, size and shape effects, and solvation contrasts. Ensemble neural networks trained on these non–linear interaction descriptors to model infinite dilution activity coefficients showed improved interpretability and mechanistic coherence, offering the first evidence that mixture‐specific interaction terms are necessary when modeling properties dominated by molecular affinity.

Finally, in their 2010 study [110], the authors returned to the initial 2006 dataset [94] and remodeled the density deviation using the new nonadditive descriptors. Using the same dataset, they also developed a model for excess molar volume. Across their three studies, nonadditive descriptors were reported to perform better for properties primarily governed by intermolecular interactions, whereas additive descriptors remained adequate, and in some cases slightly better, for composition‐dominated properties.

Taken together, these studies represent an important early framework for M‐QSPR, showing that mixture properties can be modeled from structure‐derived descriptors and that interaction‐aware terms improve predictions for non‐ideal properties. However, they also expose foundational limitations: the reliance mainly on Points‐out validation rather than Mixtures‐out or Compounds‐out strategies, the absence of a formally defined AD, and the restriction to simple binary systems, conditions far from the complexity of real multicomponent fuel mixtures.

Oprisiu et al. [72] advanced M‐QSPR modeling by again applying it to a smaller dataset of organic mixtures and this time targeting bubble‐point temperatures. The authors used fragment‐based additive and nonadditive descriptors derived from ISIDA fragments [103] and SiRMS simplexes [99]. They evaluated SVM, ASNN (associative neural network), and RF models and combined them either by simple averaging or through a stacking procedure. Differences between individual algorithms in internal validation were small, and the stacked model consistently showed the most stable behavior. Under Points‐out conditions, performance remained very high, much like in Ajmani‘s studies [94, 109, 110], with only minor variations between algorithms. In contrast, model performance showed a clear hierarchy across validation schemes: accuracy decreased under Mixtures‐out and dropped sharply under Compounds‐out. This behavior was consistent with the AD analysis, which showed that several externally tested mixtures lay outside the descriptor space of the training data, partly explaining the loss in predictive performance. This highlights the critical role of rigorous external validation and AD definition, as models that perform extremely well on mixtures composed of known components may become unreliable when applied to mixtures containing new or structurally unfamiliar chemicals. Additionally, the authors reported that in the case of the bubble point, both types of descriptors performed similarly.

M‐QSPR approaches were then used by Gaudin et al. [95, 111], Long et al. [112], and Fayet et al. [113] to model flash points of similar types of mixtures as before. Gaudin et al. investigated flash‐point prediction starting with a hybrid strategy combining pure‐compound QSPR models with thermodynamic mixing rules [111]. Motivated by the limited ability of this approach to capture nonideal mixture effects, they explored a direct M‐QSPR approach. In their M‐QSPR study [95], they used quantum‐chemical constitutional descriptors from density functional theory (DFT)‐optimized structures combined with FGCD (functional group count descriptors) descriptors and evaluated a wide set of mixing rules for constructing mixture descriptors, including linear and nonlinear expressions. Models were built using only MLR, and validation was performed only using a Compounds‐out approach. Reasonable predictive trends were obtained; however, the study underscored other recurrent limitations already seen in earlier works: performance degraded for mixtures containing underrepresented chemical families, and no AD was defined. M‐QSPR models, trained without thermodynamic constraints, offer flexibility to develop predictive concepts but are prone to diverge from accepted mixing rules due to their sensitivity to representativeness and quality of training data, hindering their generalizability. Again, this is probably due to the limited structural diversity available in the training. Long et al. [112]. approached flash‐point modeling from a different angle, focusing on electrotopological state (E‐state) descriptors aggregated through a simple additive mixing rule. In contrast to Gaudin's work [95], which relied on computationally intensive DFT descriptors, Long's study used low‐cost structural indices combined with a nonlinear algorithm (RBF‐ANN). However, their validation approach was limited to Mixtures‐out validation, meaning that the models were never challenged with unseen chemical structures. As a result, Long's model cannot be considered extrapolative in the same sense as Gaudin's Compounds‐out evaluation; moreover, both studies lacked an AD definition. Fayet et al. [113] continued the line of work, initiated in their group, with earlier Gaudin studies [95, 111], further exploring flash‐point prediction for binary mixtures using an expanded dataset and a more rigorous M‐QSPR modeling protocol. The molecular descriptors were defined as in their previous study, while mixture descriptors were generated through nine different additive, linear and non‐linear formulations. Models were developed using GA‐MLR and evaluated under a Mixtures‐out validation, because Compounds‐out validation was not possible. This time they also defined an AD using a leverage‐based Williams plot, allowing explicit identification of influential points. Their results showed improved consistency relative to earlier flash‐point M‐QSPR models [95], particularly for mixtures following regular composition‐property trends, but again, prediction errors remained higher for chemical families sparsely represented in the training dataset. As in the earlier study, their M‐QSPR models were reported to remain less accurate than the corresponding hybrid approach under the conditions investigated [111].

Zhou et al. [114] extended M‐QSPR modeling to the prediction of the superheat limit temperature (SLT) of organic binary mixtures. The authors generated thousands of molecular descriptors and used additive mixture descriptors to build GA‐MLR linear predictive models. They used three validation approaches: Points‐out, Mixtures‐out, and Compounds‐out, where the models displayed very similar performance across all, with only a moderate decline under the Compounds‐out scenario. The similar performance observed across different validation methods suggests that, within the chemical space explored in this study, SLT can be captured using additive mixture descriptors without a strong apparent dependence on explicit interaction terms. The AD was also defined using leverage‐based Williams plots, with most mixtures falling within acceptable limits, providing a clearer assessment of model reliability than in several earlier studies.

A series of studies by Wang et al. [73], Shen et al. [115], and Ni et al. [116] applied M‐QSPR methodologies to the prediction of flammability limits of gaseous binary and ternary mixtures. All three works relied on the same small dataset [117] containing less than one hundred data points, which was reused across the publications. Wang et al. [73] used quantum‐chemical descriptors combined through an additive approach and modeled the upper flammability limit (UFL) using SVM‐RBF. Their results showed a clear and expected hierarchy across the different validations, with performance decreasing again as the chemical novelty of the test mixtures increased, again highlighting the sensitivity of extrapolation to the chemical space. Shen et al. [115] revisited the same endpoint using simpler molecular descriptors and a GA‐MLR approach. However, in contrast to Wang's [73] validation strategy, their external evaluation relied only on random splits. This makes their model less informative regarding extrapolation. Finally, Ni et al. [116] extended this work by developing M‐QSPR models for both UFL and LFL (Lower Flammability Limit) using improved quantum‐chemical descriptor sets. Mixture descriptors were generated through a standard additive rule and MLR models were built and evaluated using Compounds‐out validation. Under comparable conditions, their models were reported to show better performance than those of Wang et al., suggesting that improved descriptors may partly compensate for the limited dataset size. Taken together, these studies show that flammability‐limit prediction is feasible with additive mixture descriptors and that quantum‐chemical descriptors appeared to provide improved accuracy even when coupled to linear models. All three works used Williams‐plot AD analysis, and all highlight the same recurring limitation: predictive performance is controlled far more by the limited coverage of the chemical space than by the specific modeling algorithm.

These organic‐component mixture studies suggest that additive mixture descriptors appear to be sufficient in some cases for properties largely dominated by compositional contributions, such as density or boiling point. In contrast, properties strongly influenced by specific intermolecular or reactive interactions may require nonadditive descriptors to capture nonlinear mixture effects. Across these works, the reported results suggest that model performance depends more on descriptors, validation strategy, and chemical‐space coverage than on the choice of learning algorithm, a conclusion also supported by the comparative analysis reported by Fayet et al. [118]. In particular, Points‐out validation systematically overestimates predictive ability, while Mixtures‐out and Compounds‐out schemes reveal substantial losses in performance when extrapolating beyond the training chemistry. Although AD analyses are increasingly reported, they are not applied consistently, and no standardized approach has yet been defined.

#### 3.4.2 M‐QSPR Applied to Fuel‐Component Mixtures

Saldana et al. [17] provided one of the first attempts to apply M‐QSPR to fuel‐component mixtures by modeling the flash point of hydrocarbon and oxygenate surrogate fuels. Two modeling approaches were proposed: a true M‐QSPR approach, based on mixture descriptors derived from molecular structures, and a hybrid method combining pure‐compound QSPR models with a mixing rule based on thermodynamics. In the M‐QSPR approach, Materials Studio [119] and SMARTS‐based FGCD molecular descriptors were calculated and combined using several additive linear and non‐linear formulations to obtain mixture descriptors. Models were then developed using genetic function approximation (GFA) and SVM, with validation performed under a Compounds‐out protocol. The AD definition was missing. Only the linear GFA model with Materials Studio descriptors encoding electronic, 2D electrotopological, and 3D charge‐weighted molecular‐surface information provided acceptable predictive trends, while SVM models suffered from instability due to the limited available data. In contrast, the hybrid approach was reported to demonstrate better performance. Pure‐compound properties (flash points, vapor pressures, and activity coefficients) required by the mixing rule were predicted using separate QSPR models and UNIFAC (UNIQUAC Functional‐group Activity Coefficient) [120] calculations, enabling fully predictive estimation of mixture flash points without mixture FP data for training. This method, with no theoretical limit regarding the number of mixture components, reproduced both literature mixtures and newly measured surrogate fuels (jet and diesel) with high accuracy. When compared directly with the M‐QSPR models developed in the same study, the hybrid approach was reported to deliver substantially better predictive accuracy within that study, whereas the M‐QSPR models remained limited by the narrow chemical diversity of the dataset and by the descriptive power of the available mixture descriptors. The authors emphasized that the main limitation of M‐QSPR was the lack of sufficiently diverse mixture data and the need for improved mixture‐descriptor formulations.

Jiao et al. [121] developed M‐QSPR models for the research and motor octane numbers (RON and MON, respectively) of toluene primary reference fuels (TPRF)—blends of n‐heptane, isooctane and toluene—thus applying an M‐QSPR strategy directly to gasoline‐surrogate mixtures. The structural information of each component was encoded by E‐state indices, which were then combined into mixture descriptors through a weighted sum using the mole fractions of the three components. Partial least squares (PLS) regression was used as the main modeling method, and model performance was evaluated using only a Mixtures‐out validation approach. The external predictions were reported to be highly accurate, demonstrating strong predictive performance within the constrained compositional space of TPRF blends. The AD was analyzed using a leverage‐based Williams plot, which showed that all samples fell within the defined limits, with only a single mild response outlier. For comparison, the authors also developed stepwise MLR and Scheffé‐polynomial models, using either the same E–state mixture descriptors or, alternatively, using only the mixture mole fractions as inputs, without structural descriptors. The models based on weight‐summed E‐state indices provided slightly better predictive behavior than both MLR and most Scheffé formulations. While the authors concluded that the combination of E‐state descriptors and PLS offers a simple and effective approach for predicting octane numbers of multicomponent fuels, the study remains constrained by its very narrow chemical space because it is limited to three hydrocarbons and their binary/ternary compositions. As a result, although the methodology performs well for TPRF systems, its extrapolation to broader fuel chemistries cannot be assessed.

Wang et al. [122] applied M‐QSPR modeling to the superheat limit temperature (SLT) of binary mixtures composed of hydrocarbons and oxygenated compounds representative of fuel‐relevant chemical classes. Pure‐compound descriptors were obtained from quantum‐chemical calculations, and mixture descriptors were constructed using a simple additive formulation. SVM‐RBF models were developed and evaluated using a Compounds‐out validation scheme. Under these conditions, the model showed strong external predictive ability. The AD, assessed via a leverage‐based Williams plot, indicated that most mixtures fell within reliable limits, with only a few points exhibiting high influence. Compared to earlier SLT modeling on simpler organic mixtures [114], the authors reported an overall improvement in predictive performance, which they attributed to the more advanced quantum‐chemical descriptors and the non‐linear algorithm. Overall, the study showed that SLT can be modeled effectively for fuel‐component mixtures using additive mixture descriptors.

Recently, Creton et al. [15] investigated the prediction of cetane numbers (CN) for fuel‐component mixtures and real multicomponent fuels, representing an early application of QSPR‐based methodologies directly to properties relevant to aviation fuels. Their workflow followed a hybrid strategy similar in spirit to the approaches previously explored by Saldana et al. [17] and Gaudin et al. [111], in which pure‐compound QSPR models were combined with mixing rules. In this study, pure‐compound structures were encoded using FGCD descriptors and their individual CN values were predicted using non‐linear SVM models. The CN of each mixture was then obtained by applying mixing rules directly to these pure‐compound predictions. External validation relied on repeated constrained splits: structural outliers identified by PCA were assigned to the training folds to preserve a stable AD, while the remaining data were randomly partitioned to evaluate external predictions. Several formulations were examined, including a new activity‐coefficient‐based rule, which provided the best performance for mixtures exhibiting non‐ideal behavior, particularly when oxygenated compounds were present. The framework was finally extended to real fuels represented as multicomponent (several tens) surrogates derived from comprehensive two‐dimensional gas chromatography (GC×GC) analysis, for which predicted CN values remained in close agreement with engine measurements. The study supports the idea that coupling pure‐compound QSPR predictions with an appropriate mixing rule can be more effective than direct M‐QSPR modeling.

Overall, studies focusing on fuel‐component mixtures mark a transition from methodological exploration toward properties and systems directly relevant to the fuel field. Early M‐QSPR attempts were generally reported to show more limited predictive capability than hybrid strategies combining pure‐compound QSPR with mixing rules, particularly under constrained chemical diversity. More recent works suggest that hybrid approaches can achieve reliable predictions for complex fuel properties when supported by carefully defined validation schemes and AD analyses. However, even in these more advanced studies, predictive success remains closely tied to the representativeness of the training data, while extrapolation to chemically novel fuel compositions remains constrained by the availability of reliable mixture‐level experimental benchmarks. These findings suggest that, for fuel‐component mixtures, methodological sophistication alone may not compensate for limited chemical coverage.

#### 3.4.3 Other Alternative Modeling Approaches

Beyond the previous M‐QSPR approaches based on explicitly defined mixture descriptors, several studies have explored alternative ML strategies for predicting fuel and fuel‐component mixture properties. For example, Shi et al. [123] proposed a composition–property relationship approach for predicting multiple fuel properties using ML models trained directly on mixture compositions rather than on mixture descriptors. Their dataset consisted of real fuel blends, described through compositional variables rather than explicit molecular descriptors. Several modeling algorithms were evaluated, including weighted‐average methods, PLS, GA, and a modified weighted‐average (MWA) approach that integrates both compositional and property information. Among the tested models, the MWA approach was reported to provide the best predictive performance across the investigated properties. Model validation relied on composition‐based data splits, assessing predictive accuracy within the formulation space. Under these conditions, the achieved performance was comparable to that reported by classical M‐QSPR models. However, because the models were formulated in composition space, extrapolation to mixtures containing new molecular structures or fragments could not be directly evaluated.

Hall et al. conducted a series of ML studies [23, 124, 125] for predicting key JF properties using real fuel composition data obtained from GC×GC analysis, covering both conventional and synthetic aviation fuels. Instead of defining explicit mixture descriptors, fuels were represented through compositional variables, such as hydrocarbon class and carbon number distributions, combined with pure‐compound property information when available. In their first study [23], the authors introduced a probabilistic Mean‐QSPR framework, in which pseudo‐molecular representations of fuels were constructed by averaging structure‐derived descriptors weighted by compositional fractions. Models were built using Monte Carlo dropout neural networks, enabling simultaneous property prediction and uncertainty estimation. The best predictive performance was obtained when models were trained jointly on pure compounds and fuel samples, particularly for additive or near‐additive properties such as density and net heat of combustion, while properties more sensitive to molecular interactions (e.g., flash point, viscosity, freezing point) were found to require sufficient exposure to fuel data for reliable prediction.

In a subsequent comparative study [124], Hall et al. evaluated three related modeling strategies: direct composition‐property modeling, Mean‐QSPR, and QSPR with sampling, using the same GC×GC‐derived datasets and probabilistic neural‐network architectures. Direct composition‐based models were reported to achieve the highest accuracy when sufficient fuel data were available, whereas Mean‐QSPR models showed better robustness when extrapolating within limited synthetic‐fuel datasets. Sampling‐based QSPR approaches performed well for properties dominated by carbon number effects but exhibited larger uncertainty for properties sensitive to isomeric distribution. Validation was performed using cross‐validation schemes within the available fuel datasets, focusing on predictive reliability across fuel classes rather than extrapolation to new molecular structures. Accordingly, these validations probe generalization across fuel classes/compositional domains, but do not constitute a strict Compounds‐out test at the molecular level. Finally, in their last study [125], they analyzed the impact of alkane isomeric structure on fuel properties using large pure‐compound datasets. Although no M‐QSPR models were constructed in this work, the results demonstrated that for properties such as viscosity and freezing point, isomeric effects equaled or exceed the influence of carbon number and hydrocarbon class. This finding directly supports the limitations observed in composition‐based mixture models and highlights the difficulty of capturing fuel behavior without explicit molecular or isomer‐level information.

Leenhouts et al. [105] proposed a fundamentally different M‐QSPR strategy by introducing a GNN framework explicitly designed for mixture property prediction. Instead of relying on predefined mixture descriptors or composition‐based representations, their approach constructs mixture embeddings by aggregating molecular‐level graph representations of each component through a permutation‐invariant pooling operation, weighted by the mixture composition. Molecular structures were encoded using a message‐passing neural network (MPNN), and mixture representations were generated using the MolPool architecture, which preserves both compositional information and molecular structural features. To address the limited availability of experimental mixture data, the authors adopted a transfer‐learning strategy, first pretraining the model on large synthetically generated mixture datasets and subsequently fine‐tuning it on experimental mixture data. Model performance was evaluated using a Compounds‐out validation approach, where the GNN‐based model showed strong predictive performance for several fuel‐relevant properties, including derived cetane number, flash point, and viscosity. Prediction errors were reported to approach experimental uncertainty for properties such as derived cetane number and flash point, while viscosity was identified as the most challenging endpoint. Compared to M‐QSPR and composition‐property models, this work suggests that a structure‐aware MolPool GNN can better capture nonlinear mixture behavior than traditional blending laws, particularly for oxygenated fuel mixtures.

More recently, Le et al. [126] proposed a symbolic‐regression approach for mixture property modeling that combines pure‐compound QSPR predictions with automated equation discovery to identify physically meaningful mixing rules. In their study on thermal conductivity, pure‐component properties were first modeled using ML approaches and subsequently integrated into a symbolic regression scheme to derive analytical expressions relating composition variables and component properties to mixture behavior. The method systematically explored constrained functional spaces to generate interpretable mixing equations. The resulting expressions demonstrated competitive predictive performance and good transferability across binary and multi‐component systems, including real aviation fuel samples.

Alternative modeling strategies that are not based on explicit mixture‐descriptor construction offer complementary perspectives on fuel‐property modeling. Composition‐based and mean‐QSPR approaches provide robust predictions within well‐defined formulation spaces, particularly when sufficient fuel data are available, but their extrapolative capability remains intrinsically limited by the absence of explicit molecular structure information. The GNN approach proposed by Leenhouts et al. [105] further suggests that structure‐aware models may improve prediction accuracy and generalizability compared with traditional blending laws. This study also highlights that model performance remains dependent on the quality, quantity, and chemical coverage of the available mixture data.

## 4 Summary and Outlook

M‐QSPR methodologies were reviewed with a specific focus on mixtures linked to JJFs. Across the surveyed literature, it was observed that apparent modeling performance was driven primarily by data representativeness, mixture encoding choices, and validation strategy, rather than by the sophistication of the ML algorithms employed, although nonlinear algorithms may sometimes outperform linear ones. Studies relying on Points‐out validation were generally found to overestimate predictive capability, whereas Mixtures‐out and Compounds‐out evaluations exposed the strong dependence of model robustness on chemical‐space coverage and component diversity. As a consequence, many published M‐QSPR models were found to be reliable only within narrow interpolation regimes and could not be considered extrapolative toward novel fuel chemistries without explicit AD control.

For fuel‐component systems, two broad modeling paradigms were identified. Direct M‐QSPR approaches based on predefined mixture descriptors were shown to be effective for properties dominated by additive effects, provided that the training mixtures were chemically representative of the intended application space. In contrast, properties governed by nonideal interactions and cross‐component mechanisms were more successfully addressed through hybrid strategies combining pure‐compound QSPR predictions with mixture‐level aggregation or mixing rules. In several studies, such hybrid approaches were reported to outperform direct M‐QSPR, especially when mixture datasets were limited in size or composition, highlighting the benefit of decomposing the problem into component‐level structure–property relationships and mixture‐level combination rules.

The construction of additive mixture descriptors, the application of standard regression algorithms, and the achievement of good interpolation performance within well‐sampled mixture spaces were found to be comparatively straightforward once sufficient data are available. In contrast, genuine extrapolation toward new components, new fuel families, or modified blending strategies was consistently identified as difficult. This difficulty was amplified by sparse mixture datasets, strong component imbalance, and the lack of standardized validation and AD practices. As a result, high reported performance metrics were often shown to reflect favorable data splits rather than true predictive generality.

Consequently, one of the central limitations for developing future M‐QSPR models remains the lack of open, curated, and standardized datasets. This limitation is even stronger for real fuels and SAFs, for which reliable composition‐resolved data associated with standardized property measurements remain scarce. Recent work by Abraham et al. [127] further emphasized that dataset sufficiency, functional‐group diversity, and chemical coverage are critical factors for developing robust ML models for JF property prediction. The development of benchmark datasets should therefore be considered a priority for key fuel properties. These datasets should include detailed compositional information, measurement conditions, uncertainty or repeatability estimates, mixture origin, fuel production pathway, and standardized metadata.

Within this broader data limitation, trace species represent a particularly unresolved challenge. While the presence of additives, heteroatomic compounds, and degradation products at low concentrations was frequently acknowledged as critically influential for some fuel properties such as oxidation stability, these species have generally not been modeled explicitly. This simplification was shown to be problematic, as trace species may induce nonlinear effects that are not adequately captured by conventional composition‐weighted descriptors. From an M‐QSPR perspective, trace‐species modeling remains difficult, as it requires both sufficiently resolved compositional data and modeling strategies capable of amplifying low‐fraction but high‐impact contributors without destabilizing the model. Addressing this issue will be essential for extending M‐QSPR applicability from surrogate mixtures to real fuels.

Beyond dataset development, hybrid modeling strategies were identified as a particularly promising direction for fuel‐relevant M‐QSPR. By separating component‐level structure–property modeling from mixture‐level aggregation, hybrid approaches allow chemical knowledge to be injected into the modeling workflow while maintaining data‐driven flexibility. Importantly, mixing rules should not be regarded as fixed empirical formulas only, but as adaptable components that can be learned from data when appropriate constraints are imposed. In this respect, symbolic regression [128] can be a valuable tool for discovering compact, interpretable, and physically plausible mixing rules that link predicted pure‐component properties or descriptors to mixture composition [126]. Such an approach is especially attractive for fuel applications, as it can yield transparent expressions, facilitate mechanistic interpretation, and improve extrapolation behavior compared to some M‐QSPR models.

Recent advances in structure‐aware deep learning [105] and component‐aggregation architectures [106] were also shown to complement descriptor‐based and hybrid strategies. These models were reported to better capture nonadditive effects and to improve component‐level generalization under Compounds‐out evaluation, particularly when transfer learning or pretraining was employed. However, their practical deployment for fuel modeling remains contingent on rigorous external validation, explicit AD definition, and careful uncertainty assessment, without which their apparent superiority cannot be reliably interpreted.

Finally, in parallel with property prediction, validated M‐QSPR models could also support downstream fuel‐design workflows. In particular, predictive mixture models can be coupled with optimization or search algorithms to identify candidate fuel blends satisfying several property constraints simultaneously, as illustrated by AI‐driven fuel‐mixture design frameworks [129]. Similar model‐based strategies have also been used for high‐throughput virtual screening of hydrocarbon fuel candidates, showing how predictive models can reduce the experimental search space before synthesis or testing [130]. More generally, these approaches are conceptually aligned with inverse molecular design and multiobjective molecular optimization, where property‐prediction models are used to guide the exploration of chemical or formulation spaces toward targeted properties [131, 132, 133, 134]. For fuel systems, M‐QSPR models should therefore be viewed not as replacements for experiments, but as filtering and prioritization tools that could accelerate formulation studies for aviation fuels and, more broadly, for marine, automotive, and heavy‐duty transportation fuels.

Based on the above analysis, we propose a pragmatic and incremental strategy to address mixture complexity and trace‐species effects in fuel‐relevant M‐QSPR modeling. Rather than attempting to model real multicomponent fuels directly, the proposed approach consists of progressing from simple to complex systems by first selecting a target property of high relevance and building dedicated databases starting from pure compounds. This initial stage should then be extended to low‐component mixtures, followed by a gradual increase in both compositional diversity and the number of components. Depending on the property considered, the impact of trace species should be investigated sequentially, first in pure systems, then in low‐component mixtures, and finally in more complex multicomponent matrices, where nonlinear and interaction‐driven effects are expected to become dominant. This simple‐to‐complex incremental design enables mixture effects to be isolated and learned under controlled conditions before being transferred to complex real‐fuel mixtures. Although such a strategy is highly time‐consuming, costly and property‐specific, it directly reflects the limitations identified in the existing literature, where compatible and systematically designed datasets are rarely available and are essentially nonexistent for trace‐species effects. Consequently, the construction of robust and extrapolative M‐QSPR models for real fuels is unlikely to be achieved through the direct reuse of heterogeneous literature data alone, and will instead require coordinated experimental and modeling efforts explicitly designed to support progressive exploration of mixture complexity.

## Funding

This work was supported by Association Nationale de la Recherche et de la Technologie (Grant 24/CHM/269).

## Conflicts of Interest

The authors declare no conflicts of interest.

## Supporting information

A summary table of the reviewed M‐QSPR studies, including their corresponding reference numbers from the main manuscript, is provided in the Supporting Information.

## Data Availability

The data that support the findings of this study are available from the corresponding author upon reasonable request.
