# Supplementary material for: Mixture‐Quantitative Structure–Property Relationship Approaches for Jet Fuel‐Component Blends: A Review
Source: Mol Inform. 2026 Aug 26;45(9):e70050. doi: 10.1002/minf.70050 (PMC13519380; doi:10.1002/minf.70050)
Supplement: Supplementary file 1 [file MINF-45-e70050-s001.pdf]

Table S1. Chronological summary of M-QSPR studies relevant to jet-fuel-related systems

Organic-component mixture study	Fuel-component mixture study

Publication	Properties studied	Types of mixtures	Nature of constituents	Algorithms	Molecular Descriptors	Mixture Descriptors	Internal validation	External validation	Applicability Domain
Ajmani et al. 2010 [110]	Excess molar volume (V^E)	Binary (271 mix, 4539 pts)	Organic liquids	Ensemble NN	Thermodynamic, structural, spatial and electronic (Cerius²)	Integral non-additive	Train/Validation/Test splitting	Points-out	NA
Oprişiu et al. 2012 [72]	Boiling point T (T_b)	Binary (167 train + 94 test mixtures)	Organic liquids	RF, SVM, ASNN	ISIDA fragments, SiRMS simplex	Integral additive, non-additive fragments	Points-out, Mixtures-out, Compounds-out	Points-out, Mixtures-out, Compounds-out	Fragment Control
Saldana et al. 2013 [17]	Flash point (FP)	Binary + ternary (25 mix, 287 pts)	Fuel surrogates & blends	GFA, SVM-RBF	Constitutional/geometric (MaterialsStudio), FGCD	Integral additive (linear, non-linear)	Repeated Subsampling Validation	Bootstrap Compound Out	NA
Gaudin et al. 2015 [96]	Flash point (FP)	Binary (43 mix, 435 pts)	Organics	MLR	300+ DFT-based (constitutional, topo, geo, QC) (Codessa), FGCD	Integral additives (linear, non-linear)	LOO	Compounds-out	NA
Zhou et al. 2016 [114]	Superheat limit T (SLT)	Binary (275 mix)	Alkanes, alcohols, acids, aromatics	GA-MLR	1130 descriptors (HyperChem, DRAGON)	Integral additive	Only fit	Points-out, Mixtures-out, Compounds-out	Williams plot
Long et al. 2016 [112]	Flash point (FP)	Binary (288 mix)	Organics	MLR, RBF-ANN	E-state indices	Integral additive	10-fold CV, Monte-Carlo CV	Mixtures-out	NA
Shi et al. 2017 [123]	Density, freezing pt., FP, NHOC	17 Jet Fuels	GC×GC compositional classes	WA, PLS, GA, MWA	Fraction per class (no structure)	Composition	Train/Test (Mixture Out)		Implicit (composition coverage)
Wang et al. 2019 [73]	Upper flammability limit (UFL)	Binary + ternary (78 mix)	9 gases	SVM-RBF	Quantum & electronic (DFT B3LYP/6-31G(d))	Integral additive	LOO	Points-out, Mixtures-out, Compounds-out	Williams plot
Fayet et al. 2019 [113]	Flash point (FP)	Binary (60 mix, 650 pts)	Organics	GA-MLR	~300 descriptors (Constitutional, topological…) (Codessa)	Integral additive (9 mixing formulas)	LOO, LMO	Mixtures-out	Williams plot
Shen et al. 2020 [115]	Upper flammability limit (UFL)	Binary + ternary (86 mix)	9 gases	GA-MLR	145 DRAGON descriptors	Integral additive	LOO	Mixtures-out	Williams plot
Jiao et al. 2020 [121]	RON & MON	TPRF blends (47 samples)	n-Heptane, iso-octane, toluene	PLS, MLR, Scheffé	E-state indices	Integral additive	LOO	Mixtures-out	Williams plot
Wang et al. 2020 [122]	Superheat limit T (SLT)	Binary (274 mix)	Pure fuel compounds (25 types)	SVM-RBF	12 effective DFT descriptors (B3LYP/6-31G(d))	Integral additive	LOO	Compounds-out	Williams plot
Ni et al. 2021 [116]	Lower/Upper flammability limits (LFL/UFL)	Binary + ternary (86 mix)	9 gases	MLR (ln-transformed endpoints)	Quantum/topological descriptors	Integral additive	LOO	Compounds-out	Williams plot
Hall et al. 2022 [23]	Density, viscosity, surface tension, FP, freezing pt, NHOC	Jet fuels (82 conv, 50 synth)	Fuel components	MCNN (dropout)	Mean-QSPR pseudo-structures	Integral additive	4-fold CV	Mixtures-out	Chemical space coverage
Hall et al. 2023 [124]	8 properties (incl. density, viscosity, NHOC…)	Jet fuels (75 conv, 56 synth)	Fuel components	MCNN	FGCD, Mean-QSPR pseudo-structures	Integral additive	4-fold CV	Mixtures-out	Fuel composition diversity
Creton et al. 2024 [15]	Cetane number (CN)	Pure (658) & mixtures (572)	Hydrocarbons & oxygenates	SVM-RBF/Mixing Rules	FGCD	Integral additive	5-fold CV	External real-fuel test / Mixtures-out	PCA domain
Shao et al. 2025 [24]	Density, viscosity, freezing pt, FP; NHOC	104 Jet Fuels	Fuel components	MLR, SVM, ANN, GPR, RF, GBDT	Class x C-number fractions + dist. T	Integral additive	LOO	Mixtures-out	Implicit (coverage)
Leenhouts et al. 2025 [105]	DCN, flash pt; viscosity	Fuels (up to 4 comps)	Hydrocarbons, amines, oxygenates	GNN	Molecular embeddings (MPNNs)	Integral additive (MolPool)	10-fold CV	Compounds-out	Chemical space coverage
